# Mitochondrial DNA of the Arabian Camel *Camelus dromedarius*

**DOI:** 10.3390/ani14172460

**Published:** 2024-08-24

**Authors:** Manee M. Manee, Badr M. Al-Shomrani, Fahad H. Alqahtani

**Affiliations:** 1 National Center for Bioinformatics, King Abdulaziz City for Science and Technology, Riyadh 11442, Saudi Arabia; shomrani@kacst.gov.sa; 2 Advanced Agricultural and Food Technologies Institute, King Abdulaziz City for Science and Technology, Riyadh 11442, Saudi Arabia

**Keywords:** mitochondrial DNA, arabian camel, maternal inheritance, D-loop region, genetic diversity

## Abstract

**Simple Summary:**

Mitochondrial DNA evolves rapidly, is maternally inherited, and is relatively small and compact in most animal species, making it a popular marker in molecular studies. In genomics, the study of mitochondrial DNA is important for understanding the origins, history, and adaptation of domesticated species. This review examines the D-loop region of mitochondrial DNA in Arabian camels to explore maternal inheritance, genetic diversity, and evolutionary history. It highlights significant genetic differences and adaptive traits in the mitogenome of Arabian camels. The D-loop region contains extensive polymorphisms and haplotypes, which provide insights into camel domestication and breeding processes. Comparative analyses with other camelid species reveal unique genetic signatures in the Arabian camel. Finally, this review integrates recent advancements in mitochondrial genomics, demonstrating potential applications in conservation and breeding programs to enhance the understanding and preservation of Camelidae genetics.

**Abstract:**

The Camelidae family, ranging from southwest Asia to north Africa, South America, and Australia, includes key domesticated species adapted to diverse environments. Among these, the Arabian camel (*Camelus dromedarius*) is vital to the cultural and economic landscape of the Arabian Peninsula. This review explores the mitochondrial DNA of the dromedary camel, focusing on the D-loop region to understand its genetic diversity, maternal inheritance, and evolutionary history. We aim to investigate the unique characteristics of Arabian camel mtDNA, analyze the D-loop for genetic diversity and maternal lineage patterns, and explore the implications of mitochondrial genomic studies for camel domestication and adaptation. Key findings on mtDNA structure and variation highlight significant genetic differences and adaptive traits. The D-loop, essential for mtDNA replication and transcription, reveals extensive polymorphisms and haplotypes, providing insights into dromedary camel domestication and breeding history. Comparative analyses with other camelid species reveal unique genetic signatures in the Arabian camel, reflecting its evolutionary and adaptive pathways. Finally, this review integrates recent advancements in mitochondrial genomics, demonstrating camel genetic diversity and potential applications in conservation and breeding programs. Through comprehensive mitochondrial genome analysis, we aim to enhance the understanding of Camelidae genetics and contribute to the preservation and improvement of these vital animals.

## 1. Introduction

**Figure 1:**
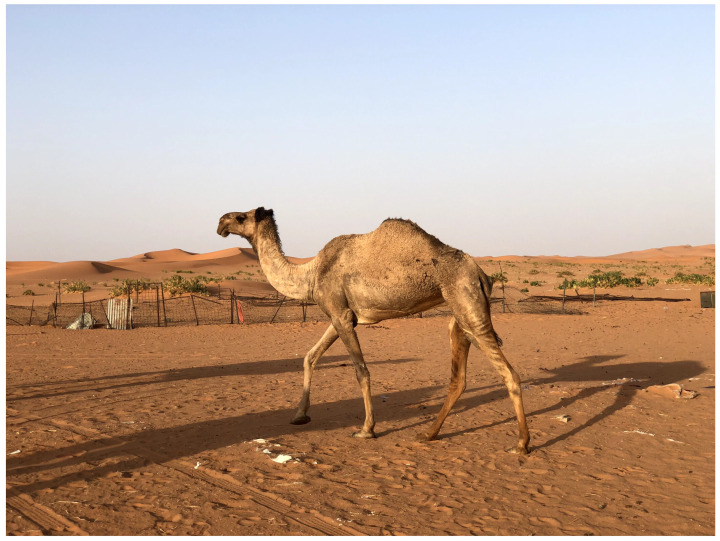
An Arabian camel in the desert of Riyadh, Saudi Arabia. Genetic exploration of these camels continues to reveal new insights into their unique adaptations and heritage.

Camelidae is a family of medium to large animals that includes several well-known domesticated species. Its geographic range spans from southwest Asia to north Africa, South America, and Australia, leading to the family’s division into two subfamilies: the Old World (Camelini) and the New World (Lamini) tribes, which diverged approximately 16.3 million years ago [1]. Originating in North America, this division was followed by the migration of the Camelini to Eurasia around 6.5 to 7.5 million years ago and of the Lamini to South America around 3 million years ago [2,3]. Although camels are not native to Australia, a large, feral population exists due to their introduction in the 19th century for transportation and other purposes [4]. Camelini includes three species (*Camelus dromedarius*, *Camelus bactrianus*, and *Camelus ferus*), while the Lamini comprises four species (wild: *Vicugna vicugna* and *Lama guanicoe*; domestic: *Lama glama* and *Lama pacos*) [5,6]. Dromedary or Arabian camels, predominantly found in the arid regions of Asia and northern Africa, constitute about 53% of the camel population of Saudi Arabia and are integral to the cultural and economic fabric of the Arabian Peninsula [7] (Figure 1). Genetic studies have identified numerous features that may be linked to the varying tolerance of Camelidae species for arid conditions with several adaptive mechanisms enabling survival in harsh climates [5,8]. Old World camels in particular exhibit a range of biological and physiological characteristics likely associated with adaptation to harsh environments, including resistance to hunger and thirst, the ability to endure fluctuating body temperatures, tolerance for high salt intake, and an immune system that produces unique immunoglobulins [9,10]. Recent genomic studies in the dromedary have uncovered loci of positive selection and immune genes that may be associated with its adaptation to desert environments [5,11].

Mitochondria contain their own distinct genome, and this mitochondrial DNA (mtDNA) is a powerful tool for studying evolutionary history and natural selection [12,13,14]. In particular, mtDNA is highly valued in phylogenetic studies due to its compact size, absence of recombination, and minimal repair mechanisms [15,16]. Maternally inherited and stable, it allows researchers to trace maternal lineages and construct phylogenetic trees [17]. Its relatively constant mutation rate is an effective molecular clock for estimating divergence times and dating evolutionary events [18], which can reveal population history, including bottlenecks and migrations. Some regions of the mtDNA are subject to natural selection, allowing the identification of adaptive changes and selective sweeps. In addition, mtDNA studies uncover geographic patterns of genetic variation, shedding light on species biogeography, speciation events, and environmental adaptation [19,20].

Variation in mtDNA has previously provided insights into dromedary populations, types, evolution, and domestication history through the identification of maternal lineages, tracing wild ancestry, and determining the geographic origins of various species [21,22,23,24,25]. The Arabian camel has unique mitochondrial characteristics that distinguish it from other camelids, including variations in key mitochondrial genes related to energy metabolism, such as NADH dehydrogenase subunit 1 (ND1), cytochrome c oxidase subunit 1 (CO1), ATP synthase subunit 6 (ATP6), and cytochrome b (CYTB) [26]. These variations likely played significant roles in the adaptation of dromedary camels to harsh desert environments. In addition, the control region or displacement loop (D-loop), which contains elements crucial for mtDNA replication and transcription, is the largest and most variable non-coding segment [27], has a higher evolutionary rate than genomic DNA [28], and hence has great utility for evolutionary studies [29,30]. The D-loop evolves faster than the CYTB gene due to higher substitution rates and is not subjected to the same selective pressures as coding regions like CYTB [31,32]. Analyzing the D-loop can also reveal patterns of genetic diversity and maternal lineage that offer insights into a species’ domestication and breeding history. Detailed studies of camel mtDNA can therefore contribute to a broader understanding of camel biology and offer potential applications in breeding programs to improve camel health and productivity.

In this review, we will explore the characteristics of the Arabian camel’s mitochondrial DNA, focusing on the D-loop region and its implications for genetic diversity and maternal inheritance. We will also discuss the role of mitochondrial genomic studies in illuminating the history of Arabian camel domestication and adaptation. Our aims are to provide a comprehensive overview of current knowledge on Arabian camel mtDNA and to highlight both the significance of mitochondrial research in camelid studies and the potential value of the mitogenome for improving breeding programs, conservation efforts, and understanding the evolutionary history of the Arabian camel.

## 2. Characteristics of Arabian Camel Mitochondrial DNA

Mitochondria are double-membrane-bound subcellular organelles that are present in nearly all mammalian cells except for a few cell types such as mature red blood cells (erythrocytes) which lack mitochondria [33]. These organelles have their own genetic material, a small, closed circular DNA molecule comprising two strands, heavy and light (H and L respectively). The compact genomic organization, nearly complete maternal inheritance [34,35], and fast evolutionary rate of the mitochondrial genome have led to its wide use as a marker for phylogenetic, phylogeographic, population genetics, and molecular evolution studies. In Camelidae, the mitochondrial genome is approximately 16 kb long (Table 1). Similar to most mammals studied, Camelidae mitochondrial genomes contain one major control region located at the $5^{\prime }$ end [36].

Few studies have specifically investigated the structure of the Arabian camel mtDNA [37,38]. In December 2015, motivated by the need to understand the molecular diversity of dromedary camels, Amer [37] compared the mitochondrial genomes of Arabian camel samples isolated from the United Arab Emirates and Morocco. The study aimed to describe and compare mitogenome features in detail, identify base differences, and offer insights into the genetic diversity within the species. The mitochondrial genome length was found to be 16,643 bp for the UAE breed and 16,665 bp for the Moroccan breed (GenBank accession no. NC_009849 and JN632608, respectively). The gene arrangement and content were consistent with other mammals, including the wild Bactrian camel. The D-loop was found to span 1222 bp in UAE camels and 1236 bp in Moroccan camels. Finally, the study identified base differences, particularly in the tandem repeat sequences of the D-loop, that could represent breed-specific variations in the Moroccan camel. All told, this study represented an initial step in understanding the genetic diversity of the Arabian camel.

**Figure 2:**
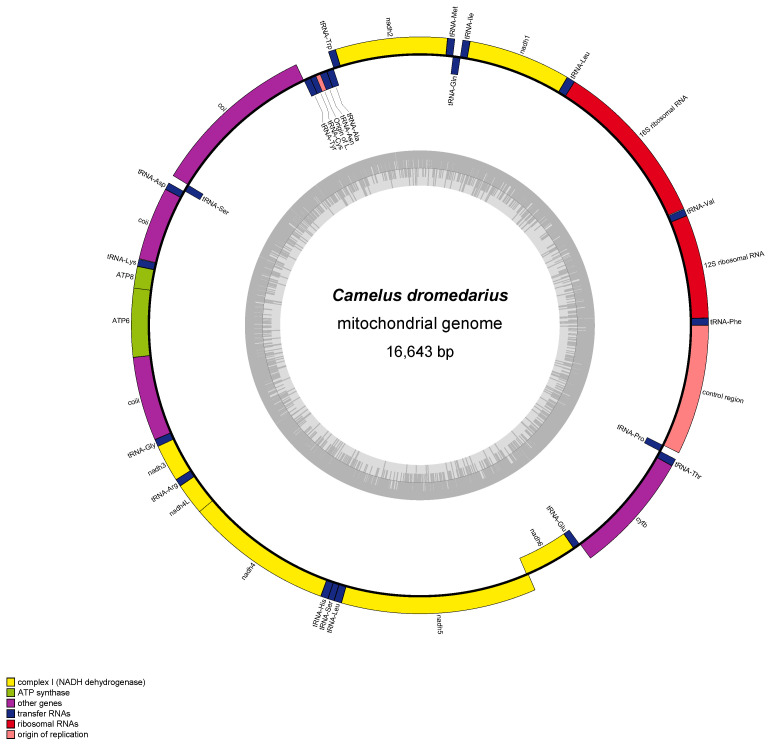
Organization of the complete mitogenome of *Camelus dromedarius* (Arabian camel), which was visualized using OGDRAW [39]. Color blocks represent distinct genes. Blocks outside the circle indicate genes on the heavy strand, and blocks inside indicate genes on the light strand. The inner gray-colored circles represent the GC content graph.

Subsequently, Manee et al. [38] conducted an extensive study on the mtDNA of *C. dromedarius* (GenBank accession no. EU159113.1), determining it to be 16,643 bp long and to comprise 37 genes, including 13 protein-coding genes (PCGs), 22 transfer RNA (tRNA) genes, and 2 ribosomal RNA (rRNA) genes (Figure 2). The D-loop is 1214 bp long, and the mitogenome additionally contains 116 bp of intergenic spacers distributed over 22 locations, with the largest spacer (33 bp) located between trnN and trnC. Base composition showed a bias toward adenine (A) and thymine (T), with 30.8% A, 27.1% T, 26.6% cytosine (C), and 15.5% guanine (G). The overall AT-skew is 0.065 and the GC-skew is −0.264, indicating a higher prevalence of cytosine over guanine. In the control and intergenic regions, the GC content is 46.7% and 51.86%, respectively. The PCGs total 11,407 bp and account for 68.5% of the mitogenome. Most genes (28) are encoded on the H-strand, while NAD6 and eight tRNA genes are encoded on the L-strand. PCG lengths range from 204 bp (ATP8) to 1827 bp (NAD5); most PCGs start with ATG, except ATP6, which starts with GTG. The stop codons include TAA (nine PCGs), TAG (three PCGs), and AGA (one PCG, COB). Overlapping sequences were identified for several genes with a total of 96 shared nucleotides; the longest overlap was 43 bp between ATP8 and ATP6, which was followed by a 17 bp overlap between NAD5 and NAD6. Notably, no introns were detected in the mitogenome. The tRNA genes ranged from 59 to 75 bp in length. Sequence alignment with other camelid mitogenomes showed 66.6% nucleotide identity with lengths varying from 16,643 bp in *C. dromedarius* to 16,084 bp in *Vicugna vicugna* [38]. rRNA genes in particular exhibited high sequence similarity among camelid species, while the D-loop showed high variability in the form of deletions and insertions.

## 3. Mitochondrial Genomes across Various Livestock Species

This section provides a concise comparison of the mitogenomes of camels with those of other animals. Camels possess a mitochondrial genome similar in size to that of other livestock species, yet they display unique adaptive genetic characteristics, which will be explored in the following sections. The average length of mitochondrial genomes in species such as cattle, water buffalo, sheep, and horses is approximately 16 kilobase pairs (Kb) [40]. Notably, the mitochondrial genome of the water buffalo lacks the tandem repeats found in other livestock species, including cattle, sheep, horses, and goats [40]. Cattle are categorized into six primary mitochondrial DNA haplogroups (T1, T2, T3, T4, I1, and I2), which vary in their geographical distribution, with the T haplogroups predominantly present in taurine cattle and the I haplogroups predominantly present in indicine cattle [41]. In goats, six haplogroups (A, B, C, D, F, and G) have been identified, with haplogroup A being the most prevalent [40,42]. The mitochondrial genomes of sheep exhibit four distinct lineages (A, B, C, and D), each with different geographic distributions [43,44,45]. Pigs demonstrate a broad range of mitochondrial haplotypes due to multiple domestication events across various regions, with some breeds showing introgression from wild boars [46]. Horses, first domesticated around 3500 BC, exhibit considerable mitochondrial DNA variability, indicating diverse origins [47], with approximately 30% of ancient horse mitochondrial diversity persisting in modern breeds [48].

## 4. Energy-Related Mitochondrial Genes in the Arabian Camel

It has been proposed that mtDNA content correlates significantly with climate variation; specifically, mtDNA content is increased in species populating cold regions [49]. Also, the mitogenome may be adapted to climate conditions in other ways; for example, thermal selection has been observed in the sequence of the ATP6 gene [50]. An earlier study by Ahmed et al. [26] investigated genetic differences between the Arabian camel and the Bactrian camel, focusing on D-loop and mitochondrial genes related to energy metabolism, namely those encoding NADH dehydrogenase subunit 1 (ND1), cytochrome c oxidase subunit 1 (CO1), ATP synthase subunit 6 (ATP6), and cytochrome b (CYTB). ND1, part of Respiratory Complex I, couples NADH oxidation and quinone reduction and hence is essential for energy transduction; dysfunctions of this protein are linked to neuromuscular and neurodegenerative diseases [51,52]. CO1, the terminal complex in the electron transport chain, prevents excessive reactive oxygen species buildup [53]. ATP6 is crucial for ATP synthesis, acting as a molecular motor in cells [54], while cytochrome b is a conserved protein with transmembrane domains and heme groups; its mutation is associated with muscle weakness and intolerance [26,55]. To examine these genes, Ahmed et al. [26] collected blood samples from 15 Arabian camels in Saudi Arabia and obtained corresponding sequences of Bactrian camels from existing GenBank database entries. A comparison of these sequences revealed 54 nucleotide substitutions (one non-synonymous) in ND1, 51 synonymous substitutions in CO1, and significant variation in ATP6, including five non-synonymous changes. However, the greatest variation was observed in CYTB, with 97 substitutions, seven of which were non-synonymous. Additionally, the D-loop exhibited polymorphisms in tandem repeats at the $3^{\prime }$ end. These genetic differences suggest that ATP6 and CYTB may play significant roles in the energy metabolism adaptations of Arabian and Bactrian camels, potentially under positive selection pressures to adapt to their respective environments. On the other hand, the high conservation of CO1 suggests strong purifying selection, maintaining its essential functions. It is also likely that some observed variations, especially in domesticated species, might result from relaxed purifying selection, where the evolutionary pressure to remove deleterious mutations is reduced.

## 5. D-Loop Region of the Arabian Camel Mitogenome

Numerous studies have examined D-loop structure in species such as cattle [56], fish [57], and birds [58]. However, most of the literature concerning Camelidae mitogenomes has focused on the overall mtDNA structure, with few studies having investigated the D-loop or its evolution. Maté et al. [59] analyzed the D-loop of the guanaco (*Lama guanicoe*), llama (*Lama glama*), alpaca (*Vicugna pacos*), and vicuña (*Vicugna vicugna*), sequencing four individuals of each species. This revealed the Lamini D-loop to consist of a 1060 bp sequence that includes three conserved sequence blocks (CSB I–III) adjacent to the tRNAPhe gene, a conserved central domain, and an extended termination-associated sequence near the tRNAPro gene. A triple repeat of a 26 bp unit was identified between CSB I and II. Notably, a 337-base pair hypervariable segment at the $5^{\prime }$ end of the D-loop, containing ten polymorphic sites, was identified by the authors as a useful genetic marker for phylogenetic and genetic diversity studies. In another study, Manee et al. [38] conducted a comparative analysis of previously sequenced camelid mitochondrial genomes of camelids. Their investigation confirmed the presence of three distinct domains: the termination-associated sequence, the conserved domain, and the conserved sequence block. In addition to reinforcing the structural organization of the control region, this study provided insights into the evolutionary conservation and functional significance of these domains across camelid species.

## 6. Signatures of Natural Selection in the Arabian Camel Mitogenome

Mohandesan et al. [1] investigated the evolutionary history of and natural selection in the mitochondrial genomes of Old World camels (genus *Camelus*) by sequencing 24 complete mitochondrial genomes from different species, including nine dromedary camels, and integrating that data with three previously published sequences. They applied the McDonald–Kreitman test, maximum likelihood approaches, and Bayesian methods implemented using MULTIDIVTIME software v. 09.25.03 to analyze mitochondrial gene diversity, detect signals of natural selection, and estimate divergence times. They also performed pairwise comparisons to determine the degree of divergence and assess the functional impacts of amino acid changes. Higher haplotype and nucleotide diversity was observed in dromedary camels compared to domestic and wild Bactrian camels, which the authors attributed to the wider geographic distribution of dromedary samples, representing three continents and seven countries; in contrast, samples from domestic Bactrian camels mainly originated from Mongolia, Kazakhstan, and Austria, and wild Bactrian camels originated from one national park in Mongolia. Purifying the selection of ND6 was observed in dromedaries, which are crucial for preventing deleterious mutations in the oxidative phosphorylation pathway; other genes did not show such selection, which was likely due to population expansion. Positive selection was suggested at specific sites in genes such as ATP8, ATP6, ND3, ND5, and ND6.

Bahbahani et al. [60] also analyzed signatures of purifying and site-specific positive selection in the mitochondrial DNA of dromedary camel populations from the Arabian Peninsula, Africa, and southwest Asia. By comparing the protein-coding mitochondrial genes of dromedaries with those of other camel species, they aimed to reveal genetic distinctions and adaptive divergences within the Camelidae family. Genomic DNA was extracted using the DNeasy^®^ Blood and Tissue kit (Qiagen, Hilden, Germany) from blood samples of seven dromedary camels, including two from Kuwait and five from Saudi Arabia; other genetic data were obtained from public databases. The authors identified genetic distinctions between the Camelini and Lamini tribes, which were likely resulting from their divergence approximately 16.3 million years ago. These included significant signals of positive selection in ATP6, COX3, ND1, ND4L, and ND5. Gene-level analyses also revealed significant signals of purifying selection among Old World camels, particularly in COX1, CYTB, ND1, ND4, ND5, and ND6. These findings highlight the roles of purifying and positive selection in maintaining mitochondrial function and driving molecular adaptation in dromedary camels.

## 7. Genetic Diversity in Arabian Camel Mitochondrial DNA

Multiple studies have utilized the gene encoding cytochrome b (CYTB) to estimate the genetic diversity of dromedary camels. In one study, the complete gene was sequenced for 48 Pakistani dromedary camels representing two breeds, Mareecha (25) and Bareela (23) [61]. This revealed five haplotypes, three in Mareecha and two in Bareela, resulting from four parsimony-informative sites at positions 137, 146, 337, and 806. Two sites were transversions (T > A and T > G) and two transitions (T > C and A > G); with regard to amino acid changes, one was synonymous and three were non-synonymous. The average haplotype diversity (*H*_d_) and nucleotide diversity (*P*_i_) were 0.83 ± 0.12 and 0.0019 ± 0.0004, respectively, indicating significant genetic diversity within Pakistani camel breeds. Xueqi et al. [62] similarly evaluated CYTB variation, specifically within an 1140 bp fragment of the gene, in 70 Nigerian dromedary camels and in published sequences from other populations with the aim of understanding the genetic diversity and evolutionary history of Nigerian camels. Sequence alignments and comparison revealed 37 haplotypes, including nine novel sequences from Nigeria, although the Nigerian population showed lower haplotype diversity compared to other countries. The authors reported evidence of recent population expansion in Nigerian dromedaries and low genetic differentiation among and within populations, which were likely due to continuous gene flow [63,64].

Legesse et al. [65] conducted a study examining the morphometric and genetic variation in eight breeds of Ethiopian camels (*Camelus dromedarius*). The study utilized mitochondrial CYTB gene sequences and six nuclear microsatellite loci to assess genetic diversity and phylogenetic relationships. While significant morphometric differentiation among the breeds was identified, the genetic analyses of CYTB sequences did not reveal monophyletic groups associated with the pastoralist-recognized breeds. The phylogenetic analyses also failed to support the identified haplotypes statistically, indicating no unique genetic lineages or significant population structure among the eight camel breeds in Ethiopia. These findings suggest that although haplotypes are present, there is a lack of statistical support for these haplotypes within the genetic data.

Satyanarayana et al. [66] investigated the within and between-breed genetic diversity in Indian dromedaries and their divergence from Bactrian camels, focusing on the ATP8 and ATP6 mitochondrial genes. ATP6 and ATP8 are crucial components, with ATP6 forming part of the proton channel and ATP8 aiding in the proper assembly of the ATP synthase holoenzyme [66]. The study also aimed to delineate the phylogenetic relationships between these camel populations and explore the presence of distinct haplogroups. The authors collected blood samples from 77 dromedary camels representing nine breeds across three Indian states (Rajasthan, Gujarat, and Madhya Pradesh) and eight Bactrian camels from the cold deserts of Ladakh. ATP8 and ATP6 mitochondrial gene sequences corresponding to *C. bactrianus*, *C. ferus*, and New World camelids were obtained from GenBank NCBI for comparative analysis and constructing the phylogenetic tree. A median-joining network was constructed to explore haplotype relationships, and phylogenetic analysis was performed using the neighbor-joining method. The study identified 15 haplotypes in the Indian dromedaries and 3 haplotypes in the Bactrian camels. Both the phylogenetic analysis and the median-joining network showed a demarcation between the Old World camels (dromedaries and Bactrian camels) and New World camelids (alpaca, llama, guanaco, and vicuña). The study identified three distinct mitochondrial haplogroups corresponding to *C. dromedarius*, *C. bactrianus*, and *C. ferus*.

The D-loop (1214 bp) was also examined in the above-mentioned assessment of Mareecha and Bareela breed genetic diversity [61]. Sequence analysis in the 48 Pakistani camels revealed 32 polymorphic sites, comprising 31 single-occurrence variable sites and one parsimony-informative site. This led to the identification of eight distinct haplotypes with four found in each breed. Phylogenetic analysis using the neighbor-joining method, supported by a bootstrap value of 1000, demonstrated that the Pakistani camel haplotypes cluster within the dromedary camel clade, which was distinctly separated from Bactrian camels. Another study by Hedayat et al. [67] investigated the diversity of the D-loop region in Iranian dromedary and Bactrian camels with the goal of enhancing the understanding and preservation of these species. The researchers collected samples from 45 dromedary camels across various regions and 29 Bactrian camels from Ardabil Province, and they sequenced a 605 bp fragment of the D-loop region in all samples. This revealed a significantly higher diversity of the D-loop in dromedaries than in Bactrian camels with 51 times more mutations in dromedaries.

Ming et al. [68] investigated the Old World camel population structure, genetic diversity, and evolutionary dynamics, including genetic relationships, historical migrations, and the impact of domestication on species genetic makeup, by sequencing the D-loop of 182 camels, including domestic Bactrian camels, wild Bactrian camels, and dromedaries. The researchers employed various molecular genetics techniques and statistical analyses to assess genetic diversity, phylogeographic patterns, and demographic history. The results identified 32 haplotypes from 156 sequences with high levels of genetic diversity among domestic Bactrian camels and dromedaries but lower diversity in wild Bactrian camels. In addition, genetic introgression between domestic Bactrian camels and dromedaries was identified based on mitochondrial DNA analysis. However, to fully confirm and characterize the extent of hybridization, further studies incorporating nuclear DNA markers are necessary. Geographic distribution influenced genetic diversity with significant variation among populations. Ultimately, the main findings highlighted the complex genetic landscape of Old World camels, demonstrating significant genetic diversity both within domestic populations and between camel types and highlighting the historical and ongoing genetic exchanges between domestic Bactrian camels and dromedaries.

Almathen et al. [24] sequenced both mitochondrial and nuclear DNA in an investigation of the genetic diversity and population structure of modern dromedaries that aimed to gain insight into their domestication, which is a subject that has long intrigued researchers seeking to understand the evolutionary history of this important livestock species. The authors analyzed ancient DNA from wild and early-domesticated dromedaries as well as DNA from 1083 modern dromedaries representing five distinct geographical regions. They sequenced 867 base pairs of the mitogenome, covering the end of cytochrome B, the tRNAs for threonine and proline, and the beginning of the control region. Contrary to the hypothesis that modern populations close to putative domestication centers retain higher levels of ancestral polymorphism, they found similar amounts of heterozygosity and allelic richness among the populations examined. This finding suggests that the genetic analysis of camels is unlikely to reveal the existence of an ancestral population or a geographic center of dispersion.

Through median-joining network analysis of the mitochondrial sequence, Almathen et al. [24] identified two haplogroups, HA and HB, containing six major haplotypes. Bayesian phylogenetic analysis supported this partition with six major haplotypes observed across the global range of the species. However, none of the six haplotypes were exclusive to any geographical region, indicating a lack of phylogeographic pattern. The researchers proposed that the two haplogroups could provide insight into the evolutionary history of dromedaries with haplogroup HA potentially representing the ancestral lineage and haplogroup HB arising due to genetic drift or selection pressures during the domestication process. Further research is needed to confirm this hypothesis and develop a deeper understanding of dromedary domestication.

Almathen et al. [24] included several samples from early-domestication times (∼1400 years ago) along with wild dromedaries from 5000 to 1000 BCE. They retrieved ancient DNA from up to 7000-year-old wild dromedary specimens from archaeological contexts in the Arabian desert, including eight wild dromedary bones from sites in the UAE and seven early-domesticated dromedary specimens from Syria, Jordan, Turkey, and Austria. The early-domesticated individuals showed no novel mitochondrial haplotypes, as most exhibited sequences identical to those of modern dromedaries, implying that both haplogroups HA and HB were already present in the Levantine herds of the fourth to seventh century CE. Different estimates of the time to the most recent common ancestor (TMRCA) of HA and HB predate the assumed period of domestication, suggesting that multiple wild maternal lineages were captured during domestication. The eight ancient wild dromedary samples presented six different mitochondrial haplotypes, with three shared with modern dromedaries and three unique to wild camels. These findings highlight the need for further ancient DNA studies, particularly from underrepresented regions and time periods, to fill missing knowledge gaps and enhance our understanding of dromedary evolution and domestication.

Abdussamad et al. [64] likewise utilized mitochondrial DNA and nuclear DNA (microsatellites) to determine if the color phenotypes recognized in Nigerian dromedaries (sand–brown, gray–white, dark brown, pied, white, brown–black, and black) are genetically distinct groups and to evaluate if those groups reflect the traditional breeding concepts of camel pastoralists in the Nigeria–Niger corridor. While their analysis provided insights into the population structure, it is important to note that coat color variation is more likely influenced by specific nuclear genes [69], which were not the focus of this study. DNA was extracted from hair collected from 75 dromedaries at the Garin Alkali livestock market in Yobe State, Nigeria, representing the various color phenotypes, and mitochondrial diversity indices were computed. The results revealed high genetic diversity with 14 polymorphisms (13 transitions and one transversion) detected in the mitochondrial DNA segment. However, the genetic data did not support distinct population structures based on coat color. Analysis of Molecular Variance (AMOVA) found that most genetic variation occurred among individuals rather than between populations, while Bayesian clustering and population structure analyses indicated that all examined populations shared a common ancestry. These findings suggest that traditional breeding concepts based on coat color do not align with the genetic data.

Two subsequent studies built on the above-mentioned work of Almathen et al. [24]. First, Alaqeely et al. [25] examined dromedary breed status and inter-population relationships in the interest of understanding genetic diversity and relationships among dromedary camel types. The researchers collected tail hair samples from 119 unrelated dromedary camels representing six camel types from Kuwait, which were then sequenced and compared to 853 publicly available sequences. Forty-eight polymorphic sites were identified, resulting in 82 distinct haplotypes among 37 different camel types. These haplotypes grouped into major haplogroups (A and B), with the B1 haplotype being predominant and present in almost all dromedary types, while A haplotypes were more abundant in African regions. The determination of haplotype and nucleotide diversity revealed a significant variability in haplotype composition, reflecting an admixture with individuals from other populations. However, Kuwaiti camel types had lower diversity compared to their Saudi Arabian counterparts, and Mezayen camel types showed a low frequency of A haplotypes, suggesting limited crossbreeding with non-Mezayen camels.

In the second study, Satyanarayana et al. [9] explored the genetic diversity and maternal origins of Indian camels, contextualizing their genetic relationships within the global diversity of the three large camel species and highlighting the importance of understanding the Indian camel population structure for better conservation and management. The authors sequenced fragments of the mitochondrial genome in blood samples from 72 dromedaries (representing nine breeds/populations from India) and eight Bactrian camels collected from the Ladakh region of India. The sequences were then analyzed for haplotype and nucleotide diversity, a median-joining network was constructed to understand genetic relationships both among Indian camels and with other camel populations globally, and AMOVA was performed to determine genetic structure and population differentiation. The results showed Indian dromedaries to feature high haplotype and nucleotide diversity, surpassing values reported in global dromedary populations [24,64]. The AMOVA analysis attributed most genetic variation to differences within populations, indicating a lack of structuring based on geographical distribution. Finally, the network topology revealed three mitochondrial lineages to exist in Old World camels: Lineage A in *C. bactrianus*, Lineage B in *C. ferus*, and Lineage C in *C. dromedarius*.

A recent study by Bardakci et al. [70] investigated the genetic variation and provided a global assessment of dromedary camels from north–central Saudi Arabia. The authors obtained hair samples from 50 dromedaries in the Ha’il Province, representing eight Saudi breeds (Majaheem, Waddah, Shageh, Zargeh, Sofor, Shaele, Homor, and Maghateer) as well as one exotic Sudanese breed (Ahmar). In addition, samples were taken from seven dromedaries in the Aydin Province of Turkey. Analysis identified 16 mtDNA haplotypes among the Ha’il Province dromedaries with high haplotype diversity (*H*_d_ = 0.817). This relatively high haplotype diversity, exceeding the range reported in other dromedary populations (*H*_d_ = 0.711–0.793) [24], suggests that the Ha’il Province may serve as a reservoir of genetic diversity. ANOVA indicated that most variance occurred within breeds. Finally, the authors performed a phylogenetic analysis incorporating the dromedary haplotype data from Alaqeely et al. [25], which included both wild and early domesticated dromedaries and spanned six geographic regions, along with the 16 Ha’il Province mtDNA haplotypes. The results revealed that the majority of samples clustered together, including both ancient wild dromedaries and the two newly identified haplotypes discovered in this study, and there was no indication of a distinctive relationship between ancient and modern Ha’il dromedaries in general, as determined by the maximum parsimony (MP) constructed tree. The authors further reported that three most common mtDNA haplotypes in the Ha’il region, which differ by only one mutational step, appear ancestral to most other dromedary haplotypes, suggesting that the Arabian Peninsula is a hotspot for dromedary diversification.

Collectively, the above-described findings provide valuable insights into the genetic diversity of and relationships among camel types, which is information that can be used to establish breed standards and conservation strategies for dromedary camels.

## 8. Conclusions

Dromedary camels have long been central to the culture and lifestyle of the Arabian Peninsula, particularly in harsh desert areas where other domestic animals struggle. This review highlights the critical role of mitochondrial DNA (mtDNA) in uncovering the evolutionary history, genetic diversity, and adaptive mechanisms of dromedaries. The unique features of the dromedary mitogenome, including its energy-related genes and the highly variable D-loop region, provide invaluable insights into the species’ adaptation to arid environments.

Analysis of complete Camelidae mitogenomes, including of dromedaries, has identified and explored signatures of positive adaptive divergence between the Camelini and Lamini tribes. In Camelini mitogenomes, there are indications of gene-wide purifying selection as well as site-specific positive selection. Given the role of mitochondria in oxygen consumption and ATP production, purifying selection in mtDNA protein-coding genes [1,60] is likely linked to adaptation to different environmental oxygen concentrations, as purifying selection eliminates deleterious alleles that impair function. The identification of such selective pressures underscores the functional importance of mitochondrial genes in adapting to extreme climates.

Understanding genetic variability within and between dromedaries is crucial for uncovering their origin, domestication, and dispersion patterns. This variability, particularly evident in the D-loop region, has proven essential for tracing maternal lineages and understanding the complex domestication process of camels. Mitochondrial DNA sequence analysis, particularly of ancient DNA, has provided key insights into the domestication and cross-continental spread of dromedaries. A recent study found relatively higher haplotype diversity among dromedaries from Ha’il Province than was previously reported in dromedary camels from five other geographic regions [24,70]. In addition, comparative analysis indicated the existence of multiple genetic lineages not defined elsewhere, suggesting Ha’il Province as a hotspot of basal diversity. Such findings emphasize the Arabian Peninsula as a crucial region in the genetic history of dromedaries and support the theory of its role as the origin of domesticated dromedaries [24]. Further identification of distinct haplogroups and haplotypes can aid in tracing the evolutionary history and domestication patterns of dromedary camels. Most prior research has primarily examined portions of the mtDNA genome, potentially overlooking the full scope of genetic diversity within the species. Extensive studies analyzing the entire mitochondrial genome of the Arabian camel remain necessary and promise to offer a deeper understanding of camel genetic diversity and evolutionary history.

Mitochondrial DNA plays a crucial role in the future of camel breeding and conservation. Identifying specific mitochondrial haplotypes linked to traits such as increased stamina and better adaptation to harsh environments allows for more informed breeding decisions, improving camel populations’ productivity and health. Moreover, mtDNA is essential in monitoring and preserving genetic diversity in captive and wild camels, which is critical for conservation efforts. The study of mtDNA in Arabian camels provides valuable insights into their evolution and genetic diversity, offering a path forward for selective breeding and conservation strategies that ensure the sustainability and resilience of camels in the face of changing conditions.

## Figures and Tables

**Table 1:** Length statistics of mitochondrial genomes for various camelid species, including *Camelus dromedarius*. The lengths were calculated for each species by analyzing all submitted mitochondrial genomes in the GenBank NCBI database (accessed on 25 July 2024). The table presents the sample size, minimum, maximum, and mean lengths for each species, providing an overview of the genetic diversity within and between species.

Species	Sample Size	Minimum Length	Maximum Length	Mean Length
*Camelus bactrianus*	135	16,398	16,856	16,617.36
*Camelus dromedarius*	125	16,375	16,738	16,620.94
*Camelus ferus*	32	16,397	16,683	16,577.78
*Camelus knoblochi*	5	16,643	16,706	16,677.80
*Lama glama*	1	16,084	16,084	16,084.00
*Lama guanicoe*	2	16,084	16,597	16,340.50
*Vicugna pacos*	4	16,379	16,680	16,596.25
*Vicugna vicugna*	2	16,659	16,680	16,669.50

## Data Availability

All the data generated in this study are included within the main text of the manuscript.
